# A qualitative study of patient (dis)trust in public and private hospitals: the importance of choice and pragmatic acceptance for trust considerations in South Australia

**DOI:** 10.1186/s12913-015-0967-0

**Published:** 2015-07-30

**Authors:** Paul R. Ward, Philippa Rokkas, Clinton Cenko, Mariastella Pulvirenti, Nicola Dean, Simon Carney, Patrick Brown, Michael Calnan, Samantha Meyer

**Affiliations:** 1 Discipline of Public Health, Flinders University, Room 2.10, level 2, Health Science Building, Registry Road, Bedford Park, 5042, Adelaide, SA Australia; 2 School of Nursing and Midwifery, Flinders University, Bedford Park, Adelaide, Australia; 3 Flinders Medical Centre, Adelaide, Australia; 4 Department of Sociology, University of Amsterdam, Amsterdam, Netherlands; 5 School of Sociology, University of Kent, Kent, UK; 6 School of Public Health and Health Systems, University of Waterloo, Waterloo, Canada

**Keywords:** Trust, Choice, Public hospitals, Private hospitals, Qualitative, Australia

## Abstract

**Background:**

This paper explores the nature and reasoning for (dis)trust in Australian public and private hospitals. Patient trust increases uptake of, engagement with and optimal outcomes from healthcare services and is therefore central to health practice, policy and planning.

**Methods:**

A qualitative study in South Australia, including 36 in-depth interviews (18 from public and 18 from private hospitals).

**Results:**

‘Private patients’ made active choices about both their hospital and doctor, playing the role of the ‘consumer’, where trust and choice went hand in hand. The reputation of the doctor and hospital were key drivers of trust, under the assumption that a better reputation equates with higher quality care. However, making a choice to trust a doctor led to personal responsibility and the additional requirement for self-trust. ‘Public patients’ described having no choice in their hospital or doctor. They recognised ‘problems’ in the public healthcare system but accepted and even excused these as ‘part of the system’. In order to justify their trust, they argued that doctors in public hospitals tried to do their best in difficult circumstances, thereby deserving of trust. This ‘resigned trust’ may stem from a lack of alternatives for free health care and thus a dependence on the system.

**Conclusion:**

These two contrasting models of trust within the same locality point to the way different configurations of healthcare systems, hospital experiences, insurance coverage and related forms of ‘choice’ combine to shape different formats of trust, as patients act to manage their vulnerability within these contexts.

## Background

The issue of trust in hospitals is of increasing importance in view of the reported decline in trust in Western healthcare systems in general [1, 2], linked to wider public distrust in a number of institutions and individuals [3, 4]. There has been a recent call for more research on trust in health care systems [5], which the authors argue is required to ‘understand, protect and restore public trust in the health care system’ (p. 1). Distrust in hospitals and healthcare professionals is problematic because it is well documented to have a negative impact on patient outcomes, a form of social iatrogenesis [6]. A Swedish study found that low levels of trust in hospitals are associated with increased risk of psychological distress [7]. After controlling for key confounders, low trust in hospitals increases the risk of psychological distress by 60 % in males and 83 % in females [7]. Patients with low levels of trust are less likely to seek or access healthcare, less likely to accept healthcare recommendations or maintain continuity of care, and more likely to avoid healthcare, including hospitals, entirely [8]. Conversely, higher trust in healthcare enhances the likelihood of return for follow-up care, increases patient adherence to therapies, facilitates health information exchange, and enables providers to encourage necessary behavioural changes [9–13]. Therefore, improving patient trust is key to improving patient access to, experience of and outcomes from healthcare.

Public concerns (and thus questioning of trust) about healthcare has been linked to evidence of inequitable allocation of resources [2, 8, 14–16], as well as high-profile medical and safety scandals [17–20]. Interestingly, many of the ‘medical scandals’ which impact public trust seem to be located in the UK and US [17], with Australia relatively unscathed by international standards. This may well have a an impact on buffering patient trust – a relative lack of large scale ‘food scandals’ in Australia, compared to UK, Europe and China, led researchers to suggest that Australian consumers have an ‘innocent until proven guilty’ attitude to making decisions on whether to trust different aspects of the Australian food system [21]. On a broader scale, the overarching declining trust in government and social administration has been linked to increased uncertainty in science, technology and expert systems [22–25], part of the reflexive modernization thesis [22]. Aupers argues that public distrust has almost become the default position, often manifest through conspiracy theory and resistance, and is the ‘cultural logic of modernity’ [26]. Bauer argues for a social science discipline of ‘resistology’ which attempts to document and understand the nature of ‘choice’ under conditions of uncertainty in late modernity, responses which are often manifest by trust/distrust [27]. Bauer argues that ‘resistance is logically and empirically a corollary of choice’ (p. 5), whereby resistance can include not making certain choices from a range of possibilities although he also argues that ‘resistance demands choice where it is denied’ (p. 5). The issue of ‘choice’ is central to the issue of trust in public and private healthcare and hospitals, since choice is an underpinning ideology in the private setting although is largely absent in the public setting.

Set within this scene of potential Hobbesian landscape, it is important to remember that despite increasing public mistrust of science and power (including medicine, hospitals and doctors), members of the public continue to access healthcare services when in need of care, excluding people who reject allopathic medicine all together . Hall et al. suggested that individuals have no choice but to trust the motives and competence of medical professions since they do not have the knowledge or skills to judge levels of expertise [28], although this lack of choice has led to a suggestion of coercive doctor-patient relationships and dependence on doctors and the medical system in general, negating the requirement of trust [29–32].

Examining trust in public and private hospitals raises important questions regarding the shifting identities and experiences of patients when accessing and viewing healthcare services as ‘systems of expertise’. Discussions of the supposed transformation of patients to consumers and even experts have become common conceptual terrain, drawing in part on notions of the reflexive modernization thesis [33–35]. The idea of making ‘choices’ and thus dealing with potential risks, is also seen as a cultural motif of ‘good citizenship’ in late modernity [36]. In this way, distrust (or at least healthy skepticism) becomes the norm [37] and indeed part of the vivacity of democracy – the ability to question [38]. The argument put forward by such theorists is that individuals have become increasingly questioning of modern institutions and ‘systems of expertise’ and consequently, access a variety of information sources in order to assess risks and choices [39]. With such a ‘horizon of possibilities’ in terms of choices and thus risks, reflexive trust (or distrust) becomes a valuable mechanism of making a decision on a course of action, thereby reducing complexity [40, 41]. Research has identified that although this thesis might hold true for some, not all people have the will, capacity or social power to weigh reflexively the risks involved in making decisions to act [42–44]. Inequalities in access to healthcare information limit some groups (e.g. older people, low socio-economic status groups) from questioning medical authority [45], referred to as stratified reflexivity – they just ‘trust’ – often referred to as generalised trust [46], habitual trust [47], assumed trust [48] or blind trust [49]. However, the explanations for ‘why’, or better yet, under what conditions, some groups do not make reflexive decisions on which to base trust or distrust remains elusive, and forms the key question for this paper.

### The context of public and private healthcare in Australia

International research has shown that funding arrangements for health systems influence trust between patient and provider [1, 17, 50, 51]. Healthcare in Australia is universal in that all Australians can receive access to public healthcare services through Medicare – government funded services funded through the taxation system. Private healthcare is also available for purchase through numerous private health insurance (PHI) companies and is encouraged by government policy. Public hospital treatment is free to ‘public patients’ (i.e. those people without PHI). However, ‘private patients’ can be treated in either public or private hospitals, both situations being paid for through their PHI. Higher income earners receive a tax penalty for not purchasing PHI [52].

In 2013, 47 % of the Australian population had some level of PHI (10.7 million) and there were 557 privately owned hospitals [53]. PHI coverage differs by age, socio-economic status (SES) and State [54]. For example, 68 % of 55–64 year olds have PHI compared to only 47 % of 18–24 year olds. PHI coverage is 79 % among the most affluent quintile of the population compared to only 33 % of the least affluent quintile, meaning that low income populations are more likely to use government-funded public hospitals. However, low SES groups generally have lower levels of trust in a range of government institutions [55, 56], linked to their vulnerabilities, disempowerment and perceived broken promises by government [31]. It therefore becomes critically important to both understand their (dis)trust in public hospitals and develop strategies to build trust [57] which is grounded in experiences of quality care and trustworthy services.

Distrust in public hospitals is of concern for the proportion of the public that cannot purchase PHI (e.g. lower earners). Additionally, distrust in public hospitals is of concern for PHI holders because in emergency situations, they will initially be cared for in public hospitals. Indeed, a large number of PHI patients are routinely treated within public hospitals for elective surgery, albeit as private patients [58]. Recent research found that private patients are treated differently to public patients in public hospitals. Private patients are assigned higher urgency, ‘jump the queues’ for procedures and are provided more medical and diagnostic procedures, irrespective of healthcare needs [58]. Whilst this may impact on longer waiting lists and more negative experiences, we do not know the impact on trust in public or private hospitals. Understanding the nature, extent and reasons for distrust in public hospitals therefore becomes paramount for both public and private patients.

### The importance of understanding patient trust in public and private hospitals

Much of the empirical and often purely quantitative literature on trust is lacking in theoretical basis [59], highlighting the need for theoretically grounded research. Indeed, the difficulty in understanding and explaining the rationality of patient trust has been identified, “medical treatment is indeed one such crucible in that the inherent risk, anxiety and suffering associated with illness are deeply imbued with meaning and thus far from generalizable” [60]. Nevertheless, Hall et al. (p. 632) argue that ‘knowing more about what conditions produce trust and distrust, and why this matters, helps to craft the structure and financing of health care delivery in a manner that supports and enhances trust’ [28]. However, there is a dearth of international research on the nature and extent of patient (dis)trust in public or private hospitals, which may be partly related to the difficulties in defining, conceptualising and thus empirically researching ‘trust’ [61].

While there has been debate regarding the definition of trust, we adopt a definition consistently used across the sociological literature: trust may be seen as “the optimistic acceptance of a vulnerable situation in which the truster believes the trustee will care for the truster’s interests” (28, p. 615), with the truster being required to ‘accept the risks associated with the type and depth of the interdependence inherent in a given relationship’ [62]. Research on trust and choice in the English NHS argued that patient trust was based on the perceived competence and reputation of doctors [51]. Some authors argue that vulnerable individuals can, in a sense, *choose* to overlook potential failings of the healthcare system [60] or in similar vein, *choose* to depend on the system [30, 63] as a strategy for minimising anxiety and managing vulnerability.

Sociological theories of trust have been explored admirably elsewhere [17, 23, 40, 46, 49, 59, 64–66], and to provide a long and detailed synthesis here would be tantamount to logorrhea. However, a short account of the key theoretical ideas that provide a backdrop for this paper are important. Trust functions as a way to reduce complexity in society [67] because placing trust (or distrust) in individuals and systems simplifies our decisions to act [40]. Trust can be placed in individuals such as doctors or nurses (interpersonal trust) and/or the systems they represent such as the hospital, the clinic or more broadly the healthcare system (institutional trust) [34]. The two types of trust are inter-related in that an individual doctor (or nurse, allied health worker etc.) represents the health system and therefore might influence trust in the system. It is entirely possible, however, for a patient to trust a doctor but distrust the underlying system. Moreover, patients can mistrust a doctor working in a trustworthy system. Interpersonal relationships can shape how patients feel about health systems and trust in the system can contribute to the development of interpersonal trust, although the way in which interpersonal trust might affect institutional trust is much less clear [50].

Trust helps people to make future decisions based on previous experience and also uses the knowledge of the past to minimise the risk of the decision [64]. Luhmann argues that trust develops with familiarity and that individuals base decisions to place (mis)trust in an individual or system on both familiarity and risks associated with decisions made for the future [64]. In the context of healthcare, individuals are likely to establish trust with known health professionals or hospitals, as their familiarity increases. Trust is likely to be enhanced in established systems known to an individual, whereby their experiences have been positive. One may hypothesise that the ‘choice’ in private healthcare would lead patients to develop a relationship with particular doctors, thereby familiarity and trust. In the context of a lack of familiarity, Luhmann argues that ‘confidence’ is required, which is semantically different to ‘trust’ [64]. Trust, for Luhmann, is an active process of making choices among different options on the basis of which option to trust [40]. However, when there are no options (e.g. in public hospitals, one cannot choose a specific doctor), it is *something other than trust* – confidence [64], dependence [30, 31], obligation [32], blind or assumed trust [48, 49] A patient may have confidence that an unknown doctor will do their best, on the basis of a familiarity with a particular hospital or the healthcare system in general. When an individual relies on confidence, there is an expectation, or at least hope, that they will not be disappointed.

It is crucial to recognise and understand the complex ways in which trust in localised relational contexts is embedded within understandings, perceptions and assumptions regarding broader systems of service organisation, professional expertise and knowledge development [40, 49, 68]. Such a systems-oriented understanding extends the analysis of trust beyond relationships between patients and doctors to include the health systems and broader social systems (e.g., economic, political, judicial) that shape knowledge and assumptions of health and healthcare [69]. In this way, (dis)trust in a hospital or a doctor may, in part, reflect (dis)trust in other social systems and institutions.

An exploratory study on reasons for purchasing PHI in Australia identified consumer trust in private healthcare (in a broad sense) as important, but did not interrogate the predictors, extent or reasons for trust [70]. Importantly, the study did not explore trust or distrust in public hospitals at all, which still remains a critically important gap in the literature [70]. Another qualitative study found that trust was lower for public healthcare, although this was limited to patients with heart disease and trust in hospital was not specifically investigated [71]. A general survey of public perceptions of healthcare in Australia did find evidence that private hospitals are trusted more than public hospitals [72], although trust in public or private hospitals was not the focus of the research and was therefore not critically or theoretically analysed. In contrast, US literature finds the opposite - private healthcare is generally less trusted than public healthcare [9] which may reflect the much larger PHI business in the US and the sub-optimal government funding of public healthcare, albeit in the process of change through ‘Obamacare’. US research argues that public healthcare systems have more open governance, are not motivated by profits and are more subject to community influence, thus increasing transparency, credibility and public trust [73]. However, no research has explored these issues within an Australian context.

## Methods and analysis

A qualitative methodology was used to explore the experiences, perceptions and observations of patients who had recently been treated within either public and/or private hospitals. Our focus in the interviews was on understanding patient trust in public and private hospitals, and the various elements of the hospital systems (e.g. doctors, nurses, cleanliness, anticipated benefits/barriers, choice etc.), stemming from the conceptual importance of both interpersonal and institution trust. Participants were sampled using a non-probabilistic, purposeful sampling method. The literature suggests that riskier medical procedures have different trust dynamics [30, 49], thus it was important to recruit patients who had experienced various levels of risk during their treatment. In addition, since ‘choice’ is linked to trust [51, 70], we aimed to access a mix of patients who undergoing urgent, semi-urgent and non-urgent procedures (this is the terminology used in hospitals), in both private and public services. To achieve this diversity, specialist doctors in both public and private hospitals agreed to provide information sheets about the study to their patients. The specialists were from Plastic and Reconstructive Surgery and Ear, Nose and Throat clinics from hospitals in South Australia. These clinics were chosen due to the likelihood of capturing potential participants who had experienced urgent, semi-urgent and non-urgent procedures. To enhance recruitment numbers, participant invitations were also published in the university newsletter.

Recruitment was challenging for this project, requiring four different recruitment methods to recruit 36 participants. The initial recruitment strategy involved three consultant surgeons, from both private and public clinics, distributing information packages to eligible participants. This recruitment method resulted in 6 participants and as a result, a more personal approach was adopted: the research assistant sat in the waiting room of two of the surgical clinics and distributed information packages to potential participants. Seven further participants were gained from this approach. The Executive Director of the Health Consumer's Alliance of SA Inc. agreed to include an advertisement in their group’s e-bulletin for three weeks, which resulted in 3 more participants. Finally, an advertisement was placed in the electronic newsletter and sent to all employees of a local university. This method succeeded in attracting 20 more participants.

The 36 participants (12 males and 24 females) ranged from 25 to 87 years in age, and included 18 participants from both public and private hospitals. Written consent was obtained prior to the interview. In-depth interviews were conducted between 2012 and 2013, at a mutually convenient time at the participant’s home, or a location of their choosing. Interviews were approximately one hour in length and were semi-structured in nature The interviews explored patient (dis)trust in the public and private hospitals in Australia and their healthcare experiences and/or sources of information that have shaped their views. Participants were asked to describe their actual experiences of being in hospitals, both as patients and as carers/family members. Although we initially sampled from public and private hospitals, assuming patients would be either ‘private’ or ‘public’ patients, we found that most participants had experiences of both settings, either as patients or carers, and could thus make comparisons based on experiential knowledge rather than conjecture.

During these interviews, the researcher probed participants’ descriptions for more detail on their perceptions of the care they received, their expectations of care, and whether these were met. This nuanced and contextually contingent approach allowed the investigators greater potential to fully understand patient narratives, as opposed to simply asking whether or not they trust their doctor, which may have just led to stereotypical factoids [74]. Interviews were audio taped and transcribed verbatim for the purpose of analysis using NVivo 8 software. Each interview was transcribed directly after the interview so that the data analysis and collection could be compared.

Three stages of analysis were undertaken: pre-coding, conceptual categorisation and theoretical categorisation. We have successfully used this analytical process [6, 21, 30, 75] and published a paper as a guide to other researchers [76]. Pre-coding provided a description of the issues or themes arising from the data, irrespective of whether they were related to the research questions. The process of pre-coding consisted of identifying words most frequently used by the participants in interviews. When the data were pre-coded, words, or sections of text, were coded using the actual words used by participants or by grouping similar words conceptually. This process was undertaken throughout the data collection process, and the initial pre-coding informed the content of subsequent interviews. Pre-coding was undertaken separately by three of the authors and discussion of coding and further refining was undertaken.

Conceptual categorisation was undertaken by grouping the initial codes into larger categories. Charmaz [77: p57] describes focused coding as ‘more directed selective and conceptual’ than the pre-coding, explaining larger bodies of text by using significant or frequent codes. This process involved an iterative process of inserting each of the initial codes into larger categories, based on their ‘semantic fit’ or the ways in which they seemed to be relating to a similar idea or issue. These initial focused codes were quite large and needed to be reduced over a number of analytical readings of the codes in order to permit sensible interpretation.

The theoretical categorisation facilitated an examination of the data from a theoretically informed perspective. This stage conceptualises possible ways that focused codes relate to each other in ways to explain a theory [77], in this case the sociology of trust. This stage was conducted by examining the focused codes with regards to theoretical and empirical literature on trust, it highlighted data that both conformed to current theories of trust and also ‘new data’. In particular, we assessed differences and similarities between participants with and without PHI in terms of their experiences and attitudes towards public and private hospitals. Importantly data that fell outside the current social theory on trust were retained and incorporated through the refinement and abductive adaption of existing theory [76]. Frequent discussions within the research team occurred to validate emerging codes.

Ethics approval for this study was obtained from the Social and Behavioural Research Ethics Committee of Flinders University.

## Results and Discussion

We have divided this section of the paper into two main parts. We provide both the results, and a discussion of them, in relation to extending the theory of trust and further understanding the nature and extent of trust in public in public and private hospitals in Australia. Firstly, we provide our analysis of the trust considerations from interviews with public patients and then we go on to explore the trust considerations from interviews with private patients. We acknowledge that we have constructed a binary categorisation of public patient /private patient to describe our participants which does not do full justice to the reality of using hospital services – ‘private patients’ often had experiences in both public and private hospitals (although predominantly the latter) although ‘public patients’ tended to only have heard about private hospitals rather than been treated as a patient in one.

### Trust considerations in public hospitals

#### Blind faith in experts

A common theme expressed by public patients was having no choice in which doctors they consulted, but also not necessarily seeing this in negative terms. Public patients, without exception, talked about the knowledge and expertise of doctors and their own relative lack of knowledge, which for them set up an innate trust, or at least faith, in the doctors. For example Darlene said

“*Trust…, for me it means that the people that are giving you advice that- could ultimately determine your life or death potentially, sensing, and it is just a sense, it’s not based on anything other than a sense, sensing that you are in good hands, that the outcome may not go well but at least everybody’s doing everything they possibly can to give you the best level of care and the best chance. So for me the pivotal moment of that was when I was told I would need to go into emergency surgery which would require a general anaesthetic that under the circumstances may not be ideal but that was the only option and that sense of ‘oh crap things aren’t going particularly well here’. We’re getting down to minutes now, not hours but I just had this sense of ‘well, you know what, I can’t physically change the outcome myself. I’m in good hands. I just have to trust that this is going to go okay’. And had that sense from the staff as well, that I was in the best hands. It’s intangible to describe really I guess”* (female, 38, public).

This extended quote highlights a number of the key issues in this section of the paper. This participant talked about the uncertainties and intangibles involved in trust (“*it is just a sense*”), the vulnerabilities patients face when in medical emergencies (“*ultimately determine your life or death potentially”*), the lack of choice one has during these circumstances (“*I just have to trust*”), but also a pragmatic acceptance of the situation (“*I can’t physically change the outcome myself. I’m in good hands”)* and a sense of optimism that she was in “*the best hands*”.

Similar to previous research [51], it was difficult for participants to differentiate between ‘trust in doctors’ and ‘trust in the hospitals’, since the doctor was the flesh-and-blood representative of the hospital. Indeed, Giddens’ [34] notion of the ‘access point’ articulates this, whereby individuals invest (dis)trust in the system (i.e. hospital) through their inter-personal interactions with the representatives of the system (i.e. doctors or other staff).

Lillian simply said, “*As for hospitals, we really go in with blind faith”* (female, 72, public), whereas another participant specifically linked the asymmetry in doctor-patient knowledge to his faith in doctors, “*Well I’m not trained medically so I’m taking a lot of what they say on faith”* (male, 47, public). This ‘acceptance’ of asymmetric knowledge and expertise is reminiscent of Parsons’ ideas about the sick-role [43, 78]. However, the unreflexive part of ‘blind faith’ sits in contradistinction with Luhmann’s theory of trust [64], which presupposes that trust is built on experience and familiarity. On this experiential basis, a decision is made, amongst a variety of decisions that ‘could’ have been made, to trust a particular doctor. However, in the context of public hospitals, participants seem to trust ‘doctors in general’ and then in specific circumstances, transfer this trust to individual doctors who they rely on for care and treatment. This ‘dependence’ has been found in other contexts of health care [2, 30, 63]. Whilst there has been a movement around ‘patient expertise’, patient-centred care and ‘shared decision making’ [79–81], there have also been questions around whether choice ‘really’ exists for vulnerable, marginalised and/or low income groups [29, 82, 83].

In addition to the numerous statements about ‘blind faith’, there was the added temporal dimension mentioned by participants. The sheer urgency of being taken into an emergency department meant that actively seeking information on which to base trust, whether a public or private patient, become impossible. In this context, patients had little option other than to ‘go with the flow’, as outlined by Jodie:

*“Yes because you’ve got faith in the hospital trauma or emergency department and the time, you don’t have time to Google them or whatever so you just have to trust them. They’d have to be highly skilled to be in that environment. I know things go wrong and people get ramped and they’ve got to sit in the ambulance and stuff but I think they’re really very skilled in that department”* (female, 39, public).

Jodie made an assumption that the staff were ‘highly skilled’ and used this as the basis of trust, even when ‘things go wrong’. A number of public patients made comparisons with hospitals in the past or in resource-poor countries or the US, stating how ‘fortunate’ they are to even have a publicly funded system. The following quote illustrates this point, stating that they lived in a metropolitan area where they actually have hospitals, in comparison to being in a ‘desert’, and they used this comparison to justify and rationalise their ‘blind trust’. Interestingly, Colin also talked about health care professionals being ‘in charge’ of him and being ‘in their hands’, symbolising a shifting of responsibilities:

*“We’re in their hands aren’t we really because we’re in a system where – you know, we’re not lying out in the desert and we don’t have to come up with a tourniquet and bite on a piece of wood, do we? We’re in their hands and they’re medicating us. Generally yes, I trust the people that are in charge of me”* (male, 48, public).

### Pragmatic acceptance

An over-riding theme from public patients was their acknowledgement of the various ‘problems’ associated with public hospitals, and the publicly funded healthcare system in general. There was a mix of personal experiences and exposure to negative media reporting, predominantly about long waiting lists and ramping (patients remaining in ambulances outside emergency departments, sometimes for long periods of time, until beds become free in the emergency department). The following quote from Michael describes a participant’s pragmatic acceptance (i.e. recognise the problem as intractable) of the ‘failures’ in the publicly funded health system and their unwillingness to criticise or challenge it:

*“I’m a realist and as long as you have human beings in anything something’s going to fail sometime because that’s human nature. You are going to get the mistakes…. you just pray that it’s not you or one of your relatives or whatever else. No, it’s a good system. It’s a faulted system but it’s a system that we’ve got and it’s a system that I’d be happy with”* (male, 51, public).

This quote, and a number of others in the analysis, shows the knowledge of the health system and its failings, but also a recognition that humans in the system (e.g. doctor, nurses) are trying to do their best. There was a palpable sense with all public patients of both respect and sympathy for healthcare professionals working in public hospitals, which led them to fervently defend the public system. Michael also had a sense of hope when he said he would *“pray it’s not one of my relatives that are in the hospital when the mistake is made”.* For this and other participants, the base-level trust seems to be in the medical/hospital system, which may relate to a trust in hospital bureaucracy, doctor registration, medical training, quality and safety systems or medical research and drug discovery. For Giddens [34], the hospital would be conceptualised as the ‘access point’ within which trust is won or lost, although Giddens recognises the durable trust in ‘systems’ and the more fallible trust in ‘individuals’, “*although everyone is aware that the real repository of trust is in the abstract system, rather than the individuals who in specific contexts “represent” it, access points carry a reminder that it is flesh-and-blood people (who are potentially fallible) who are its operators*” (p. 85).

For a number of the public patients, pragmatic acceptance was not just for ‘non-urgent’ elective care, but carried over into their experiences of emergency care. Linked to both ‘blind faith’ and a lack of choice, participants talked about the fact that they ‘had’ to trust the hospital staff. Darlene’s recollection of her experience of emergency surgery in a public hospital is an example of both accepting the relative chaos of emergency departments and investing trust when there is arguably no choice. Prior to the previous quote from Darlene where she talked about *‘just trusting’*, she had been talking about the reasons she was rushed into hospital and the ‘chaos’ of the system when she arrived, which highlight the perceived lack of choice but to ‘just trust’:

*“Yes. I did have a few moments in that process going ‘oh oh’ – I remember one of the staff commenting to me ‘you seem remarkably calm’ and I just remember saying to her ‘well, I don’t know what other choice I have at this point to be honest. I could flap and be in quite a mess but that’s not going to serve any of us well at this point so it is what it is and let’s get on with it’ really”* (female, 38, public).

Another public patient, Emma, used to pay for PHI, but is no longer able to afford it. She talked about the benefits of private hospitals over public hospitals, and would certainly prefer to have PHI again. However, now that she ‘has’ to use the public hospitals, she exhibits a form of pragmatic acceptance:

*“So many people like myself that just can’t afford private cover that they need to use the public system. I guess there’s not much you can do really because there’s just getting more and more people, isn’t there, now that use the system? …..You can’t get it right for everyone, it’s just not possible, we’re human”* (female, 44, public).

The following public patient, Christina, talked about a variety of negative experiences in emergency department, but despite these negative experiences, tried to rationalise the situation and still talked about having trust:

*“Well in the XXXX [name of hospital] I was actually told by the person treating mum, she was so flat out that had no time to do the obs …. and I have no medical …I haven’t got any medical training…. It was not ethical…but it’s only because they were stretched to the max and you could understand the pressure…”* (female, 85, public).

### Sustained optimism

Public patients were uncomfortable being seen to complain about or criticise public hospitals, and when they made seemingly negative comments in interviews, they countered these with excuses or justifications. All of the public patients in our study ‘justified’ potentially negative elements of public hospitals through what we argue is sustained optimism (i.e. the individual doctors are still trying to do their best under difficult circumstances), for example:

*“Okay, they might be overflowing with people but if it was a serious thing they would eventually get round to you. Fortunately I’m not out in the country areas and it’s a different scenario there. I’m in a city so – yeah”* (male, 75, public).

Another participant talked about the media stories of ‘ramping’ (treating patients in ambulances outside hospitals because the emergency departments are full), which could be seen in very negative terms. However, this participant recognised the problems inherent with this practice and assumed that the paramedics would just take him to another hospital and he would trust their judgement and the care at the un-named hospital he would be taken to:

*“XXX [hospital name], yeah, not this one, or the - you know, you hear the stories about all the ambulances banked up down here so they might send me to somewhere else, you see, and I’d say well good on them because they’ve got me where they could quickest and I would accept whatever treatment was available”* (male, 64, public).

In this respect public patients seek ways to maintain their trust, possibly because they have no fall-back position (i.e. in the free, allopathic medical system). If public patients were to distrust, they may force themselves into an uneasy state of existential anxiety [84], not knowing where to turn or which knowledges to privilege – an apparently generalised state in late modernity known as both ‘era of insecurity’ [85] and ‘culture of anxiety’ [86], neither of which might be regarded as salutogenic [87] or eudemonic states. We argue that in such circumstances, trust is sustained through optimism. With respect to public hospitals, if optimism was removed, then patients may simply be left with complaint and negative feelings, potentially leading to distrust. However, distrust may be too unsettling in public hospitals because there are little if no alternatives for health care. The sustained optimism therefore becomes a necessary strategy whereby mistakes or lack of funding are viewed through the lens of optimism.

Jeff had repeated negative issues while in a public hospital, but kept qualifying/justifying the problems he experienced while in hospital. He was a ‘private patient’, but his condition was dealt with in a public hospital. He expressed being let down by the system post-surgery, being forgotten about, and being left to fend for himself. He notes how “packed out” the hospital was and talked about his internal struggle to justify both the competency and yet inadequacy of the system that let him down: “*I think we are very lucky to have this system and that we’ve got it at all…they were just so busy and I do understand…there was nothing they could do and it was just going to be one of those things”* (male, 64, private). He mentioned repeatedly how busy and chaotic the hospital was and that he did not know what was happening to him or his care, but accepted that it was just ‘how it is’: “*it just seemed like it was unorganised and chaotic and busy and all of those things at once…you’re just bombarded with people walking in, walking out…it was just a blur”.* As soon as he commented on the difficulties incurred, he went on to defend the doctors and nurses working in the hospital: “*you do very much become part of the system there. I think it’s very much that and you can understand why, because there is so many patients, after being in there and seeing the patients that come through, I do have a lot of sympathy for them…and the nurses were fantastic”.* Interestingly, he did criticise the PHI company whose ‘red tape’ made it difficult to claim from. He is bearing a certain amount of guilt for still costing the system money due to his delayed recovery: *‘I’m costing the public system. I’m costing everyone.’*

### Trust considerations in private hospitals

In non-emergency health scenarios, a division between public and private participants interpretations/consideration of trust appears: For the private patients, the central foci related to a choice of hospital and physician/surgeon. Private patients had a number of explanations for their trust in private hospitals, relative to public hospitals. Choice is a concept not really up for offer in public hospitals:

“*I think the private system, for me it gives me flexibility to be sceptical and to make choices amongst who does the work, that’s probably the advantage. In the public system I think you take what you’re given” (male, 47, private)*

### Choice and reputation

Keith made a link between patients being able to choose their doctor in private hospitals and the doctors themselves needing to develop and sustain a positive reputation, an informal league table, with the winners having more patients and thus more income:

*“.. some of the doctors in the public system are not up to scratch, but they wouldn’t last in the private system…if you get a bad reputation you won’t get people referred to you because the old boys’ club, you’d call it, or the group, you’d be a reject really quick if you were no good. That said I’ve seen a couple of guys in private practice – not treating me fortunately – but who have been a bit less than ideal. Then again that’s the reason I wouldn’t go to see them even though I was asked if I’d be referred to them because I did know that they were unsuitable” (male, 56, private)*

By implication, Keith’s remark about specific doctors being “a bit less than ideal” and “unsuitable” suggests the capacity and ability to criticise the performance of doctors in private hospitals, and ultimately to choose a different doctor. This sense of choice was not seen as possible in public hospitals, whereby any criticism tended to be both accepted and excused, thereby retaining the status quo of trust.

A number of private patients talked about the importance of reputation for doctors in private hospitals, often talked about as ‘pressure to perform’. Reputation of surgeons was also an important determinant of trust in the UK when ‘choice’ was brought into the English NHS [51]. This perceived competition within and across private hospitals was perceived to improve the quality of care and made the choice of doctor easier through reputational trust. However, the corollary was that the lack of competition in and between public hospitals does not force doctors to constantly assess and improve their quality *vis a vis* their peers or ‘customers’:

*I guess me personally, yeah, I’d probably always go for a private setting for any kind of surgery. Yeah so I must, part of me….the private doctors, they’re so dependent on their reputation so if they are really bad then it affects their livelihood whereas in public maybe there’s not that pressure on them. They’re not as well known. When you turn up, you get who you get. (female, 39, private)*

Contrary to patients in public hospitals who felt unable to criticise their doctors, Julie felt able to criticise her care while being treated as a public patient (with a work cover injury) within a private hospital. Implicit within her belief is also that she can complain in private hospitals whereas as a public patient she cannot:

*“I would rather go to xx public hospital than xx private hospital again…I mean Dr X doesn’t know any of that because I never told him. I think he’d be horrified if he knew the way I was treated that morning…If I’d been a private patient and it would have come out of my pocket I would have really said something” (female, 56, public)*

George paid for PHI and was both reserved and sceptical about how doctors could help him. Although he said he did not distrust the medical profession, he was certainly cautious and sought alternative therapies rather than consult doctors. He described trust as an objective criterion of a doctor’s abilities, and was something that can and should, in his view, be assessed by patients and demonstrated by doctors. In this way, George talked about something akin to ‘earned trust’, as opposed to less reflexive concepts such as blind or assumed trust. He talked about making a decision whether or not to ‘submit’ himself to the doctor’s recommendations, suggesting an active engagement and possibly an internal struggle with considering whether or not to trust a doctor and follow their advice:

*‘Well I’ve seen doctors that to my mind, they just don’t really have the experience or the depth of understanding to understand the problems that I’ve got….. but I need to have someone who understands where I’m coming from before I can have the level of confidence in them to submit to their direction on this…I want these people to really demonstrate to me they understood what was going on with it and that they knew how to fix it before I’d submit to it(male, 47, private).*

### Personal responsibility resulting from their choice

There is a concept amongst some private patients that they have to take responsibility for their own health and their own decisions, a form of shared care and patient centred-care. Luhmann [64] argued that trust means choosing one action (consent to surgery) in preference to another (have a second opinion or choose a different doctor), in spite of the possibility of being disappointed by the actions of the trusted person [64]. The following quote from Randall shows a want to take on responsibility for deciding which doctors to consult, and in so doing, to also take on the blame if their trust was misplaced. Luhmann [40] argued for the importance of this form of ‘self-trust’, and also for the internal attribution of blame and self-doubt:

*“It’s not so much the full trust I just think it’d be silly – yeah, I guess it’d be silly to just place your full trust in someone else when it’s your health. My health is my responsibility. It’s my responsibility to seek out a GP if I’m sick and if I don’t then it’s my own fault. If I go to a GP and just take their word for it and it’s wrong, well, it’s not their fault, it’s my fault because I didn’t do my own homework. They’re not perfect” (male, 35, private).*

Natasha had PHI and suffered a traumatic loss which left her with, “*a huge loss of trust in the system*” (female, 50, private). On the basis of this loss of trust, she took-on more responsibility for her own health care as a result of her bad experiences in the system *“I can’t trust anyone to be doing the right thing because they didn’t in the past so it’s got to be up to me”.* When confronted with a major health issue, Natasha found a way back into the system by, *“taking control… it’s up to me…I knew I had to be prepared and steer the ship in going back into the health system”.* This interview was interesting as she created a way to cope with a system she had completely lost trust in. Her strategies involved working out the best way to develop a relationship with the individuals in the system in order to get the information she required in order to make an informed decision. Instead of ‘blindly’ trusting the system, this participant had developed a format of critical, conditional trust [51, 88] in order for her to extend her control over her care and manage her vulnerability.

## Discussion and Conclusion

This was a qualitative study in a single Australian state, predominantly drawing participants from a relatively limited number of hospitals and sub-specialities within hospitals, and also from responses to advertisements. Research is required to broaden the range of participants, in terms of geographical areas and specialities they have accessed, and also their experiences with healthcare services. We did not specifically sample participants on the basis of having made complaints against hospitals or doctors, or indeed people who have ‘rejected’ medical science in favour of complimentary therapies. These would be fruitful areas of enquiry in order to understand the ‘limits’ of trust and what happens when trust is not repairable. Additional research would also be valuable from the supply side of the medical equation – studies with healthcare professionals, complaints managers and policy makers in public and private healthcare organisations in order to understand how they attempt to develop, maintain and extend trust with patients.

Participants in this study articulated ‘trust’ in the hospital they used (public or private), which leads to an overall finding that both public and private hospitals are ‘trusted’ by their patients/consumers. This finding certainly surprised us, since we assumed that trust in public hospitals would be lower for all participants. A previous Australian survey which found higher trust in private hospitals sampled a broad cross-section of the Australian public and could, by using a questionnaire survey, only ask very broad questions [72]. Our decision to undertake in-depth interviews and specifically sample participants directly from public and private hospitals may account for the more contextually specific and nuanced findings in our study. We did not ask people to make judgements about a hospital system they had not used, since this may have invoked sensationalist, media-driven ‘factoids’ [74], rather than responses based on their actual experiences and reflections on attending public or private hospitals.

We drilled-down into the preconditions and nature of ‘trust’ that was seemingly apparent in both public and private hospitals, and found differences in the reasoning for trust in the two systems. Public patients talked freely about their lack of choice of doctors within public hospitals, their understanding and ‘pragmatic acceptance’ of the system - ‘warts and all’ – and also their optimism that doctors were doing their best to treat them within the constraints of the publicly financed system. Public patients articulated a kind of ‘forced or resigned trust’ in the doctors, albeit limited in scope given their lack of choice, and were willing to accept and sometime excuse or justify the ‘problems’ of the system in order to maintain their trust. This ‘problem’ may have serious implications for patients with chronic conditions requiring regular care within public hospitals. The lack of perceived agency to reflexively ‘choose’ to (dis)trust public hospitals questions, in a Luhmannian sense, whether public patients are indeed ‘trusting’ at all [64]. Meyer and Ward [30] argue that in situations of medical emergency, patients have no time or choice to make decisions about (dis)trust, they are simply dependent on the doctors and the medical system. However, our analysis reveals that even when public patients have time and often reasons to consider (dis)trust, the lack of alternatives for free health care creates a similar dependence. Participants did not exhibit ‘blind trust’ since they were highly aware of financial problems within the public system, but they experienced doctors providing the best care they could under difficult circumstances. These doctors were thus viewed as benevolent ‘knights’ [89] who were perceived as deserving of patient trust because they were ‘doing their best’.

For private patients, the centrality of choice meant that private doctors were described as being required to ‘pick up their game’ in order to increase or sustain their reputation, thus enhancing quality and trust. They made comparisons with doctors in public hospitals who, apparently lacking in incentives around competition, correspondingly were perceived to provide lower quality care. ‘Private doctors’ were seen to be ‘doing their best’ in order to retain or improve their reputation, which in turn translated into patient trust. These views reflect the neoliberal assumptions on which private healthcare is built, whereby competition in the ‘marketplace’ is assumed to drive up the quality of health care [90]. These private patients conceived of medical professionals as knaves, whose self-interests were oriented in line with quality care [89].

Private patients also talked about the process of choosing a doctor bringing personal responsibility to them as individuals – they assessed various forms of information in order to choose a doctor and imbue them with trust, and if this trust was broken by the doctor behaving poorly, the onus and blame were partly felt by the patient. However, in such circumstances they had the ‘luxury’ of being able to then choose another doctor, unlike people using public hospitals who may be able to get a second-opinion, but unlikely to be able to ‘choose’ the doctor. Nevertheless, the centrality of the need for self-trust [40], trusting their own decisions to trust a particular doctor, was apparent for private patients although much less so for public patients.

The participants in this study presented a rather black-and-white picture of the conditions for trust in public and private hospitals and doctors. There was no recognition in the interviews that a large proportion of doctors in hospitals in Australia undertake both public and private clinics, and therefore the ones ‘picking up their game’ in a private hospital one day, are likely to be ‘doing their best in an underfunded system’ within a public hospital the next day. This is certainly an area in need of future research in mixed health care systems like Australia. The two different models for the conditions of trust articulated by public and private patients point towards the influence of healthcare systems on the ‘dimensionality’ of patient trust [88]. However, also apparent within our data were the agency and creativity of trusters in constructing positive expectations [60] as a means of managing vulnerability amidst uncertainty. Trust in such circumstances is best considered as a ‘forced option’ [66], but different social systems can be seen as forcing trust in different directions. Whilst this paper has begun to explore the trust considerations in public and private hospitals, these issues are likely to become even more important as national governments grapple with ways to provide universal and equitable publicly-funded healthcare in difficult fiscal climates and under policies of austerity.
